# Flow Increase Is Decisive to Initiate Angiogenesis in Veins Exposed to Altered Hemodynamics

**DOI:** 10.1371/journal.pone.0117407

**Published:** 2015-01-30

**Authors:** Volker J. Schmidt, Johannes G. Hilgert, Jennifer M. Covi, Nico Leibig, Johanna O. Wietbrock, Andreas Arkudas, Elias Polykandriotis, Cor de Wit, Raymund E. Horch, Ulrich Kneser

**Affiliations:** 1 Department of Plastic and Hand Surgery, University Hospital Erlangen, Friedrich-Alexander-Universität Erlangen-Nürnberg, Erlangen, Germany; 2 Department for Hand-, Plastic- and Reconstructive Surgery, BG Unfallklinik Ludwigshafen, Universität Heidelberg, Heidelberg, Germany; 3 Department for Physiology, Universität zu Lübeck, Lübeck, Germany; Centrum Wiskunde & Informatica (CWI) & Netherlands Institute for Systems Biology, NETHERLANDS

## Abstract

Exposing a vein to altered hemodynamics by creating an arteriovenous (AV) shunt evokes considerable vessel formation that may be of therapeutic potential. However, it is unclear whether the introduction of oscillatory flow and/or flow increase is decisive. To distinguish between these mechanical stimuli we grafted a femoral vein into the arterial flow pathway of the contralateral limb in rats creating an arterioarterial (AA) loop (n = 7). Alternatively, we connected the femoral artery and vein using the vein graft, whereby we created an AV-loop (n = 27). Vessel loops were embedded in a fibrin filled chamber and blood flow was measured by means of flow probes immediately after surgery (day 0) and 15 days after loop creation. On day 15, animals were sacrificed and angiogenesis was evaluated using μCT and histological analysis. Mean flow increased from 0.5 to 2.4 mL/min and was elevated throughout the cardiac cycle at day 0 in AV-loops whereas, as expected, it remained unchanged in AA-loops. Flow in AV-loops decreased with time, and was at day 15 not different from untreated femoral vessels or AA-loop grafts. Pulsatile flow oscillations were similar in AV-and AA-loops at day 0. The flow amplitude amounted to ~1.3 mL/min which was comparable to values in untreated arteries. Flow amplitude remained constant in AA-loops, whereas it decreased in AV-loops (day 15: 0.4 mL/min). A large number of newly formed vessels were present in AV-loops at day 15 arising from the grafted vein. In marked contrast, angiogenesis originating from the grafted vein was absent in AA-loops. We conclude that exposure to substantially increased flow is required to initiate angiogenesis in grafted veins, whereas selective enhancement of pulsatile flow is unable to do so. This suggests that indeed flow and most likely wall shear stress is decisive to initiate formation of vessels in this hemodynamically driven angiogenesis model.

## Introduction

Hemodynamic forces are important regulators for maintenance, growth and regression of the vascular network [1]. Under healthy conditions, endothelial cells are highly metabolically active, but mitotically quiescent [2,3]. Increases in blood flow or the change from laminar to turbulent flow acts as an angioinductive signal onto the endothelium which is mediated by intra- and extracellular signalling cascades [1]. While many studies focused on these molecular processes and contributing transduction processes within the cell [4,5], less is known about the mechanical forces that induce angiogenesis. Most of these studies investigated vascular responses to altered hemodynamics, such as pulsatility or mechanical load, *in vitro* [6]. *In vivo* studies that were focused on embryonic vessel formation revealed that vessels with high blood flow exhibit few or no tip cells and that an increase of flow is accompanied by an induction of splitting angiogenesis, so called intussusception [7,8]. In the mouse and chicken embryo, impaired perfusion of the vascular plexus in the yolk sac compromises the remodeling of capillaries into arteries and veins [9,10]. Shear stress, pressure and oxygen were considered to be responsible for the maintenance of identity genes in developing arteries of the vascular plexus in the chicken embryo yolk sac [9]. Moreover, blood flow is crucial for inducing sprouting events in the aortic arch involving the activation of the shear stress sensitive gene kfl2 in zebrafish embryos [11]. Watson and colleagues demonstrated fundamental differences between hypoxia-driven and developmental angiogenesis in the developing zebrafish embryo and pointed out that blood flow is required for angiogenesis in response to hypoxic signaling but is not required for normal vessel patterning [12].

Previously, we established a model to investigate hemodynamically-driven angiogenesis *in vivo* [13,14], which is distantly related to the early work of Erol and Spira [15] and was further modified by Mian, Tanaka and Morrison [16–18]. In this model a grafted venous vessel is interposed between the femoral artery and vein in the rat to create an arteriovenous (AV) shunt. The grafted vein is placed as a loop into a fibringel-filled chamber that is implanted subcutaneously. Being exposed to a considerably increased flow with pulsatility that is comparable to or even exceeded values observed in arterial vessels [19], remodeling and development of an increased smooth muscle layer hyperplasia was observed in the grafted venous vessel wall [20,21]. Subsequent sprouting of capillary-like structures into the periphery of the chamber was detected predominantly originating from the venous graft [14], and after two weeks the chamber is for the most part interlaced with a 3-dimensional vascular network [22,23].

In this model the expression of the gap junction forming protein connexin43 (Cx43) was specifically increased in the endothelium of the grafted vein [19]. Cx43 is a member of a larger family of connexin proteins of which four are expressed in cells of the cardiovascular system (Cx37, Cx40, Cx43 and Cx45) [24–27]. However, only Cx40 and Cx43 have been suggested to be involved in angiogenesis [9,28]. Previously, enhanced Cx43 expression was found in response to shear stress, mechanical load, or stretch in vitro [29] and all of these mechanical stimuli are modified in the grafted vein used as an AV shunt.

Because this angiogenesis occurs without application of angiogenic factors [30,31] it is tempting to speculate that it is primarily elicited by altered hemodynamics acting onto endothial cells or the vessel wall in the grafted vein [19]. We hypothesized that enhanced flow, but not enhanced pulsatility or pressure-induced wall tension is required to initiate angiogenesis in the grafted vein. Therefore, we investigated flow characteristics, expression of Cx43, and formation of new vessels in venous grafts, either interposed as an arteriovenous (AV) shunt or engrafted into the arterial flow pathway (arteroarterial grafting, AA) thereby in the latter preserving downstream microvascular resistance. The AA setting supposedly prevents flow increases (expected to occur in the AV setting), but exposes the grafted vein to arterial pressure and enhanced wall tension as well as to arterial flow pulsatility in a manner comparable to the AV scenario.

## Material and Methods

### Experimental design

Experiments were performed in 2–4 month old male Lewis rats (n = 34) with a body mass of 280–448 g obtained from Charles River Laboratories (Sulzfeld, Germany). All procedures were in accordance with the German Animal Welfare Act and approved by the Institutional Animal Care and Use Committee of the Regierungspräsidium Mittelfranken (54–2532.1–34/09). Rats were anaesthetized using isoflurane to interpose a femoral vein harvested from the contralateral thigh either as an arteriovenous loop (AV, n = 27) between femoral artery and vein or as an arterioarterial loop (AA, n = 7) within the femoral artery flow path. The constructed loop was embedded in a fibringel-filled chamber as described [19] and placed subcutaneously for 5 or 15 days to allow new vessel formation as previously described [14] before angiogenesis was investigated.

### Surgical procedure

**Fig 1:**
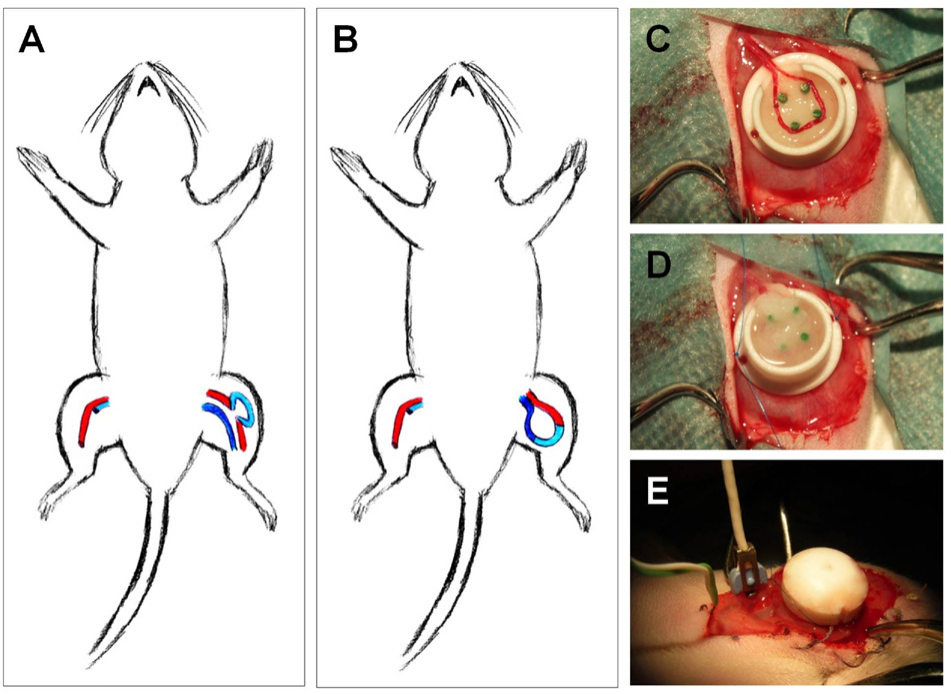
Experimental procedure and chamber containing the venous graft. The femoral vein was obtained from the right leg and was interpositioned as a graft in the left leg in the arterial flow pathway (arterioarterial [AA] loop, A) or the graft was used to create an arteriovenous shunt (AV loop, B) between femoral artery and vein. In both settings, the grafted vein loop was placed in an isolating teflon chamber (C) that was filled with a fibrin clot and stitched to the fascia of the underlying muscle (D). An ultrasonic transit-time flow probe was placed around the vessel entering the chamber for measurements of blood flow (E).

Surgical procedures were performed in all rats by the same investigator using a surgical microscope (magnification 20x, OPMI IFC, Carl Zeiss, Jena, Germany). Animals underwent inhalation anesthesia (5% isoflurane; Baxter, Vienna, Austria) and received tramadol for pain relief (7.5 mg/kg i.v.; Tramal, Grünenthal, Aachen, Germany), benzylpenicillin-streptomycin (0.5 mL/kg i.m.; Veracin-compositum, Albrecht, Aulendorf, Germany) and heparine (80 IU/kg Liquemin, Ratiopharm Ulm, Germany). After midventral incision of the medial right thigh, the femoral vascular bundle was exposed, dissected and separated from the proximal pelvic artery downstream to the bifurcation of the femoral artery at knee level. A femoral venous graft with a length of 20 mm was harvested from the contralateral hind leg and interposed as a loop either between artery and vein (arteriovenous loop, AV) or within the femoral artery flow path of the left thigh (arterioarterial loop, AA) using 11/0 sutures (Ethilon, Ethicon, Norderstedt, Germany). In some experiments the diameter of the veins was measured before explantation and after grafting and reestablishing flow through the vein. Thereafter, the entire loop was either placed subcutaneously (only for rtPCR, n = 6) or embedded into an isolation chamber (n = 28) made of Teflon by placing it carefully around the four plastic tubes (Fig. 1). The Teflon chamber was fixed onto the underlying adductor fascia (Prolene 6/0, Ethicon, Norderstedt, Germany) before the wound was closed by cutaneous sutures (Vicryl 3/0 and 4/0, Ethicon, Norderstedt, Germany). Animals were kept at a 12 h dark/light cycle with free access to standard chow (Sniff) and water before and during the experiment. At the end of the experiment animals were sacrificed by intracardial injection of a combination of embutramid, mebezonium and tetracain (15 ml/kg; T61H, Intervet) under deep anesthesia (5% isoflurane).

### Blood flow measurement

Blood flow was measured continuously at the femoral vessels (artery and vein) during initial surgery and at the loop before transfer into the fibrin filled chamber for at least 10 min using a microcirculatory flow probe (transit time flowmeter, TS420, Transonic, Ithaca, USA). Data were stored with Data Acquisition Software Windaq (Dataq, Akron, USA) at 240 Hz. This procedure was repeated 15 days after implantation to assess blood flow in the AA and the AV loops. For standardization, vascular resting tone was abrogated by topical superfusion of papaverin (1mM, Paveron N, Linden Arzneimittel Vertrieb, Heuchelheim, Germany) and maximum blood flow was analyzed. Cardiac cycle dependent oscillations of blood flow (flow amplitude) were determined as maximal flow difference during a cardiac cycle. Animals exhibiting no flow in the constructed loop at day 15 were excluded from further analyses (n = 2 in AA loop, n = 1 in AV loop).

Data were imported into Excel sheets (Microsoft, Redmond, USA) for further analysis. A macro written in Visual Basic determined peaks of blood flow at every systole and the corresponding miminum before this peak. The difference between these two extremes was taken as blood flow amplitude. Furthermore, we determined diastolic flow by calculating mean flow over a period of 0.05 s before the start of the systolic flow increase.

### Histological and micro-CT analysis

For visualization of the newly formed vessels in the constructed loop the distal descending aorta was cannulated using a 24 gauge catheter immediately after the animal was sacrificed. 100 mL of warmed (37°) isotonic salt solution containing heparin (100 IE/mL) were injected, followed by infusion of 30 mL India ink solution [50% v/v India ink (Rohrer&Klinger) in 5% gelatin and 4% mannitol]. In some animals micro-CT analyses were performed. Hitherto, 20 mL of warmed yellow Microfil (MV-122) containing 5% of MV Curing Agent (both obtained from Flowtech Inc., Carver, USA) were applied instead. To allow histological analyses, constructs were explanted and immediately fixed in 3.5% formaldehyde, dehydrated and embedded in paraffin.

Histological slices of 5 μm thickness were obtained in standardized planes (500 mm proximal and distal of the central plane) perpendicular to the longitudinal AV loop axis. After staining with hematoxylin and eosin according to standard protocols, slices were visualized using conventional microscopy (Olympus IX81, 10x magnification, Olympus Corporation, Hamburg, Germany) and analyzed using commercially available software (Olympus cellSens dimension, Olympus Corporation, Hamburg, Germany). Micro-CT scans were acquired on a high resolution, cone-beam micro-CT scanner developed at the Institute of Medical Physics, University Erlangen, Germany (ForBild). Algorithms used for enhancing the images and further technical specifications are described in detail elsewhere [32]. The evaluating operator was blinded to the experimental groups.

### Quantitative PCR (qPCR)

Subcutaneously implanted venous AV (n = 4) and AA (n = 3) loop grafts were harvested 5 days after surgery during isoflurane anesthesia, instantaneously frozen in liquid nitrogen and stored at -70°C until later analysis. A part of the femoral vein, which was dissected and reintegrated into the venous limb of the circulation by microsurgical techniques served as control (sham operated group, n = 3). This vessel was treated otherwise similar as those vessels interpositioned between artery and vein or between artery and artery. Frozen tissue was disrupted using a mortar and pestle, total RNA extracted by TRIzol (Invitrogen), and purified with RNeasy kits (Qiagen, Hilden, Germany). After reverse transcription (Omniscript RT Kit, Qiagen), quantitative PCR (qPCR) was performed using iQ SYBR Green Supermix (BioRad). Connexins and GAPDH as the reference gene were detected using the following primers:

Cx43: 5’-CGTGCCGCAATTACAACAAGCA-3’ (forward)

and 5’-TGGAGTTCATGTCCAGCAGCAA-3’ (reverse),

GAPDH: 5’-ACCACCCAGCCCAGCAAGGATA-3’ (forward)

and 5’-GCCCCTCCTGTTGTTATGGGGTCT-3’ (reverse).

The comparative CT method was used for quantification of gene expression.

### Statistical analyses

Graphs were plotted with Graphpad Prism (GraphPad Software, Inc., La Jolla, USA). Excel with its built-in Visual Basic Macro was used for calculation of blood flow parameters. Data are presented as means ± SEM and compared using unpaired t-test. For more than 2 groups analysis of variance (oneway ANOVA) followed by the Bonferroni post hoc test was used. All data were distributed normally. Differences were considered significant at a corrected error probability of p<0.05.

## Results

### Basic data

Complications caused by anesthesia or surgery were absent in all animals (n = 34). There were no signs of wound dehiscence or infection in either group. A fibrous capsule surrounding the chamber was evident in all animals at the stage of explantation. 4 rats have undergone serous fluid puncture during the first week following surgery (n = 3 in AV, n = 1 in AA). At the time of explantation patency of the AV loop and absence of thrombosis was verified in all animals due to a successful perfusion of Ink-solution and / or via direct blood flow measurement using flow probes. 3 animals (n = 1 in AV, n = 2 in AA loop) exhibited no flow and ink staining was absent most likely due to loop thrombosis. These animals were excluded from further analyses. In all remaining animals the perfused main vessel axis was macroscopically visible through the matrix. The explanted matrices exhibited no signs of fibrinolysis or degradation. Diameter of femoral veins before explantation was 457 ± 17 μm (n = 2). Veins were distended similarly after interpositioning as an AV graft (727 ± 6 μm, n = 3) or as an AA graft (749 ± 9 μm, n = 3, p = 0.12 vs. AV).

### Hemodynamics in the loops

**Fig 2:**
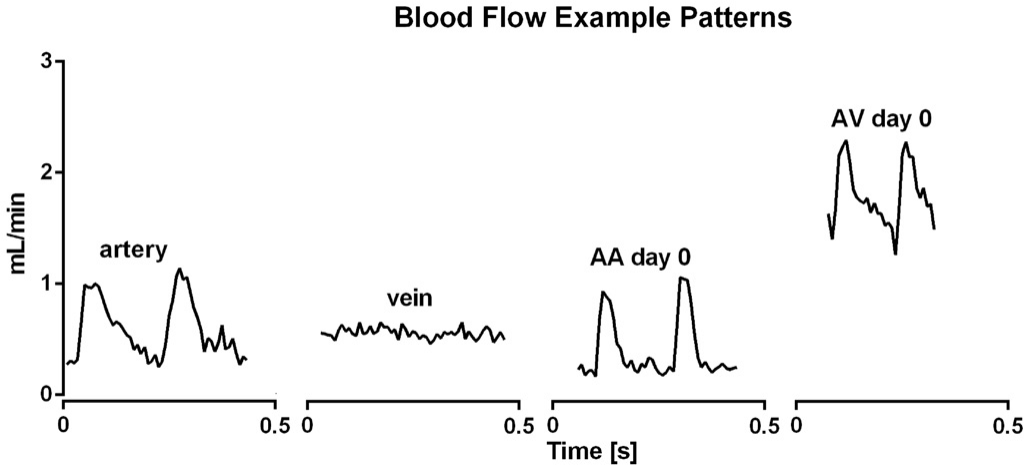
Representative examples of blood flow measurements. In the untreated femoral artery, but not vein, blood flow varies at a frequency (~300 min^-1^) that is to be expected for heart rate in anesthetized rats. Blood flow through AA and AV loops created by interposition of a vein graft was similarly pulsatile at a frequency consistent with heart rate at day 0. However, a more continuous increase in flow is only observed in AV loops, in which an arteriovenous shunt was created.

**Fig 3:**
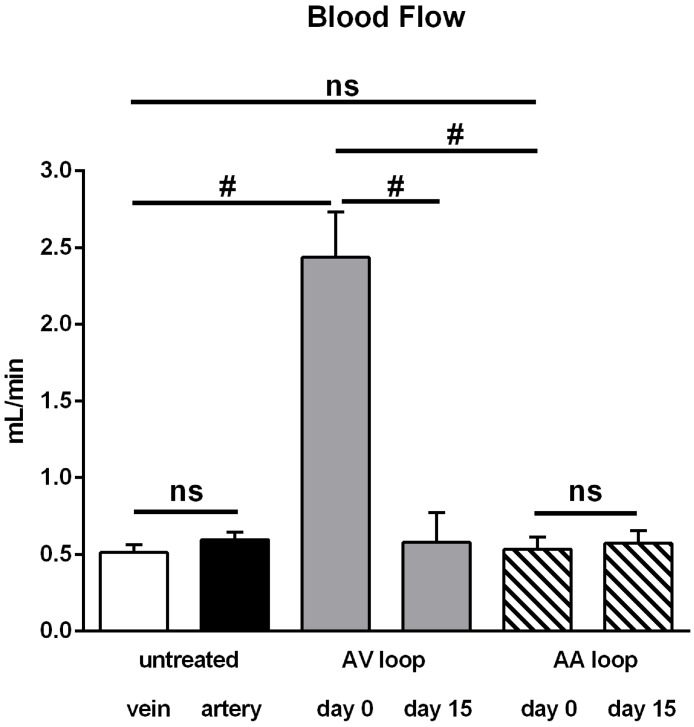
Mean blood flow in untreated vessels, arterioarterial (AA) and arteriovenous (AV) loops. In untreated femoral arteries and veins (each n = 28) flow was similar. Flow through a loop created by interposition of a vein graft into the arterial flow pathway (AA) was not different from untreated vessels immediately after surgery (day 0, n = 7) and remained at this level until explantation (day 15, n = 4). In contrast, mean blood flow through AV loops was substantially enhanced at day 0 (n = 27), but attained the level of untreated vessels at day 15 (n = 11). All values are mean+/-SEM. #: *P*<0.001 vs. other groups.

**Fig 4:**
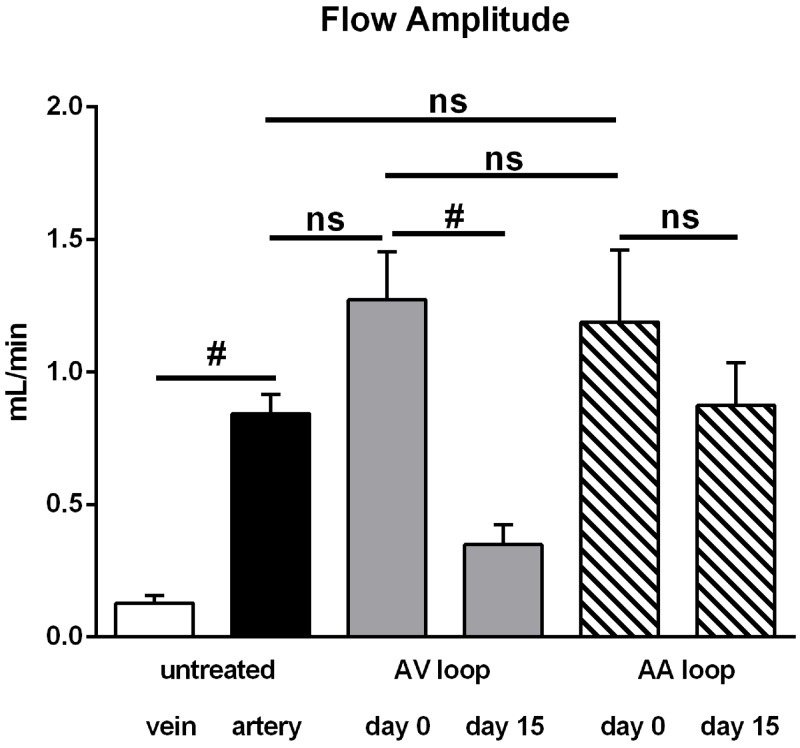
Flow amplitude in untreated vessels, arterioarterial (AA) and arteriovenous (AV) loops. Flow amplitude was calculated as the difference between maximal flow and the corresponding minimum value determined before from data sampled at 240 Hz. The amplitude was neglible in untreated femoral veins and significantly larger in untreated femoral arteries. Immediately after surgery (day 0), flow amplitude in AA and AV loops was at the level of untreated arteries. While the amplitude was significantly decreased in AV loops at day 15, it remained unaltered in AA loops. All values are mean+/-SEM, number of observations (n) is identical to Fig. 3. #: *P*<0.05.

Hemodynamic data were obtained using microvascular flow probes in each rat (n = 34) at high speed collection (240 Hz). Fig. 2 shows representative examples of blood flow during cardiac cycles. Mean blood flow amounted to 0.60 ± 0.05 mL/min in untreated arteries and was similar in untreated veins (0.51 ± 0.05 mL/min, Fig. 3). As expected, flow was more pulsatile in arteries than in the accompanying vein, which was calculated as the difference between maximal and minimal blood flow during a cardiac cycle (Figs. 2 and 4).

**Fig 5:**
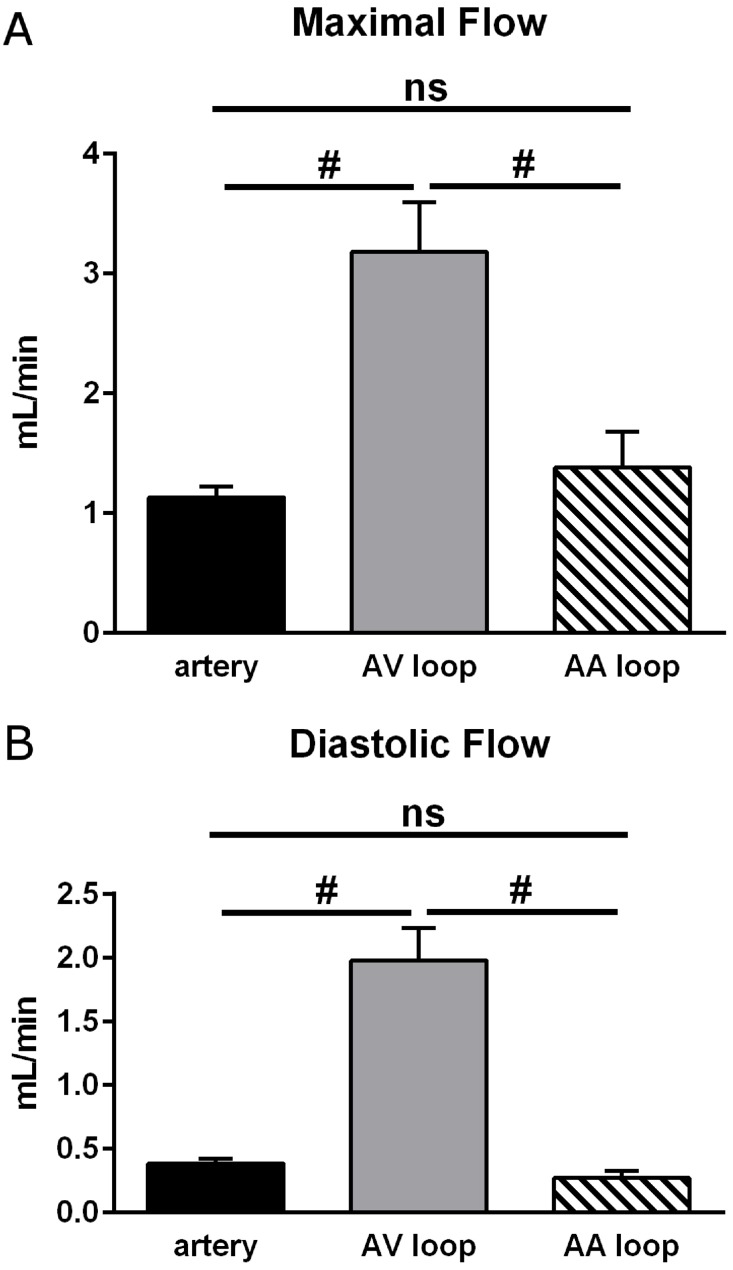
Maximal and diastolic flow in arteries, arterioarterial (AA) and arteriovenous (AV) loops at day 0. Maximal flow (A) occurred during systole and diastolic flow (B) was determined as the mean value before the distinct flow increase indicating the next systole. Flow in AV loops was strongly enhanced at both times indicating that flow increased throughout the cardiac cycle. In contrast, neither maximal nor diastolic flow was enhanced in AA loops compared to arteries. Considering the similar diameter of veins transplanted either as AV or AA graft (for values see text, basic data) wall shear rate is accordingly elevated in AV loops throughout the cardiac cycle compared to AA loops. All values are mean+/-SEM, number of observations (n) is identical to Fig. 3. #: *P*<0.05.

Interposition of a venous graft creating an arteriovenous shunt (AV) caused a dramatic increase of mean blood flow (2.44 ± 0.30 mL/min) through the grafted vein immediately after surgery (day 0). Despite the large and significant 4.7-fold increase in blood flow compared to an untreated vein or artery (Fig. 3), flow amplitude did not increase in the grafted vein of the AV loop compared to untreated arteries although a tendency was observed (Fig. 4). Fig. 5 displays maximal and diastolic flow at day 0 and demonstrates that maximal flow is tripled and diastolic flow quadrupled in AV loops compared to arteries. Thus, the strong increase in blood flow in AV loops is due to a flow increase throughout the cardiac cycle and only in a limited fashion related to an enhancement of the flow amplitude (example trace in Fig. 2 at the right). However, flow amplitude was enhanced compared to untreated veins (Fig. 4). As observed previously, the enhanced flow in the AV graft was not long lasting and at day 15 when the rats were sacrificed flow was not different from flow in untreated vessels (Fig. 3). At this time point, flow amplitude was not significantly different to untreated arteries but larger than in untreated veins (Fig. 4).

In contrast to the AV shunt model, blood flow was not enhanced after interposition of a venous graft into the arterial flow path (AA loop: 0.53 ± 0.08 mL/min) following surgical procedure (day 0), which was virtually identical to the mean flow in untreated femoral vessels (Fig. 3). Considering the diameters of grafted veins (measured in a few experiments, see above) the mean wall shear rate was substantially reduced in AA loops (~ 210 s-1) compared to AV loops (~ 1080 s-1) at day 0. The mean blood flow at day 15 was unaltered and maintained its initial value (0.57 ± 0.08 mL/min). However, flow amplitude in AA loops was similar to that in AV loops and thus also enhanced compared to untreated veins but not different from untreated femoral arteries. Maximal and diastolic flow determination revealed that flow in AA loops was not different from arteries and strongly reduced throughout the cardiac cycle compared to AV loops at day 0 (Fig. 5). Interestingly, the flow amplitude in AA loops did not decline after 15 days as was observed in AV loops (Fig. 4).

### Angiogenesis in the grafted vein

Fifteen days after loop implantation angiogenesis was visualized in 12 rats (AA n = 5, AV n = 7) by means of histological staining of cross sectional slices and additionally in 5 rats (AA n = 2, AV n = 3) using micro-CT analysis.

**Fig 6:**
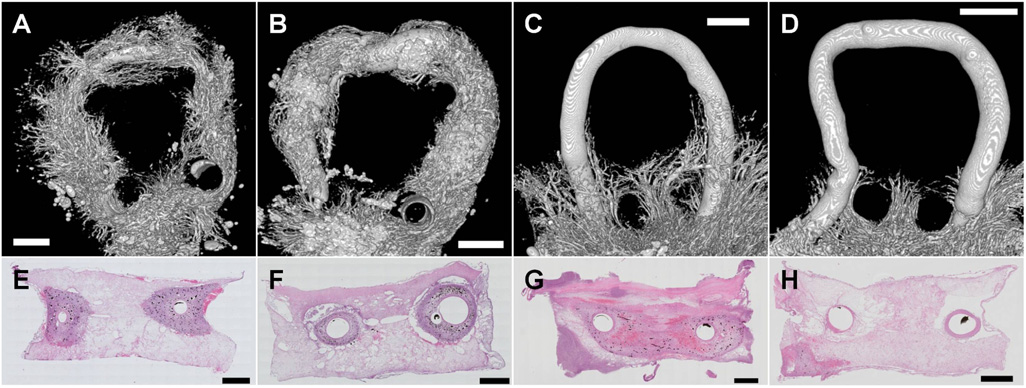
Vessel formation in arterioarterial (AA) and arteriovenous (AV) loops at day 15. Micro-CT images were acquired after injection of appropriate dye shortly after sacrifizing the animal and explantation of the loops. Representative examples of reconstructed images of AV loops (A,B) show extensive formation of new vessels arising along the overall length of the grafted vein. In marked contrast, newly formed vessels in AA loops (C,D) were only present at the entrance and exit of the loop in and out of the chamber (bottom), but not arising from the grafted vein within the loop (top and middle). Histological slices of AV loops (E,F) reveal new vessels in close proximity to the grafted vein which are stained with black ink that was injected into the aorta before explantation verifying perfusion. The lack of angiogenesis from the graft in AA loops was observed also in histological preparations (G,H). Newly formed vessels were absent (H) or in a greater distance from the graft (G). Scale bars: 1000μm.

Luminal and homogenous vessel formation was evident in micro-CT images (Fig. 6A, B) in the fibrin matrix surrounding the grafted vein along its entire length, if it was grafted between artery and vein (AV). This was also observed in HE stained cross sectional slices (Fig. 6E, F). Angiogenesis occurred without addition of any angiogenic factors as previously described in a similar loop model [19]. In contrast, in AA grafts newly formed vessels were only visible in the area of the matrix at the entrance and exit of the loop forming graft (bottom in Fig. 6C, D). These vessels were also detected in cross sectional slices (Fig. 6G, H), however mostly not in close proximity to the grafted vessel. The 3-dimensional micro-CT analysis revealed that luminal sprouting was never detected from the grafted vein along its loop in AA (upper part in Fig. 6C, D and S1, S2 Videos.

### Cx43 mRNA expression

**Fig 7:**
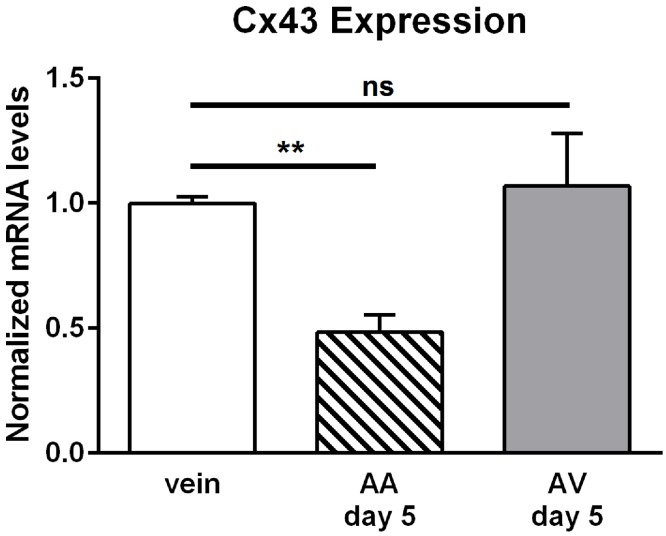
Expression of connexin43 (Cx43) at the mRNA level. Expression of Cx43 was quantified at the mRNA level in explanted loops (AA n = 3; AV n = 4) and in veins that were dissected and reintegrated into the venous limb circulation using quantitative PCR. Values in untreated veins (vein, n = 3) are taken as 1 after normalization to the expression level of GAPDH. At day 5 after implantation, Cx43 gene expression was reduced in AA, but not in AV loops compared to sham treated veins. All values are mean±SEM, ** *P*<0.01 unpaired *t* test, Bonferroni corrected.

We previously demonstrated that Cx43 mRNA expression and Cx43 protein abundance were enhanced in veins grafted between artery and vein [19]. Therefore, we investigated Cx43 mRNA expression in the venous graft following arterioarterial interposition. Compared to sham operated control veins Cx43 expression was decreased at the mRNA level in veins grafted into the arterial blood path after 5 days, but not in veins grafted as an AV shunt (Fig. 7).

## Discussion

Hemodynamic factors are important stimuli to initiate angio- and arteriogenesis. The present study demonstrates that enhanced flow itself is the most important factor to induce angiogenesis in veins. By grafting a vein either between an artery and vein (AV) or into the arterial flow pathway (AA) we were able to distinguish between different flow conditions exerted onto the grafted vein, namely selective enhancement of pulsatile flow or a rise in pulsatile flow combined with flow increase throughout the cardiac cycle. If the vein was exposed to enhanced pulsatile flow without flow increase (AA model) angiogenesis was absent. Only if additionally flow increased (AV model) vessel formation arising from the grafted vein was evident. Because diameters of the grafted veins were similar in both scenarios the continuously enhanced flow in the AV setting suggests that the high wall shear stress acting onto the venous endothelial cells is the key mechanical force to initiate angiogenesis. Currently, it remains unclear if concomitant increases in oscillatory shear stress are also required, but solely enhancing oscillations is without effect.

Hemodynamically induced angiogenesis can be elicited by creating an AV shunt embedded into a matrix that allows endothelial sprouting. Previously, we have shown in a rat model that AV shunt associated angiogenesis preferentially occurs at veins exposed to altered hemodynamic conditions [14] and can be enhanced by the application of fibrin gel-immobilized VEGF and bFGF [13]. In contrast, subjecting arteries to such conditions in a graft model results only in limited vessel formation [14]. Therefore it was tempting to speculate that exposing a vein to enhanced flow oscillation acts as a main contributor. However, flow also increases in this model [19]. In the present study, we exposed veins only to increases in pulsatile flow by grafting the vein into the arterial flow pathway, which does not introduce a large pressure drop and thus flow through the graft remains constant. Under these conditions angiogenesis originating from the grafted vein was virtually absent. In marked contrast, simultaneous flow increases by positioning the graft between artery and vein induces considerable vessel formation as we have shown previously using this model and was also described by others [33–36]. Inflammation due to the surgical procedure, denervation of the transferred vessel segment, handling of the graft and composition of the matrix were similar in both procedures and can be excluded as being causal for angiogenesis. Another mechanical force that changes for the engrafted vein is the enhanced transmural pressure by exposing the vein to arterial pressure levels. This may lead to vessel distension and increased wall tension. However, these changes prevailed in both approaches, AA and AV loops, in a comparable manner as verified by similar diameters of the veins after grafting. Additionally, arterial pressure was very likely similar in both scenarios because shunt flow through the AV graft was only 2.4 mL/min which is less than 5% of the cardiac output in rats (amounting to 60 to 75 mL/min, [37]). Therefore it is highly unlikely that arterial pressure is reduced due to the AV shunt flow. Thus, wall tension, being determined by pressure and diameter, is unlikely to act as the decisive stimulus for the angiogenesis observed only in the AV model. This view is further supported by the fact that (if anything) wall tension will decrease in the AV model along the grafted vein as pressure decreases due to the resistance at high flow loads. In contrast, pressure in the AA scenario decreases not along the grafted vein but at the physiologic location, the downstream resistance vessels (arterioles).

Hypoxia is often referred as a crucial inductive factor for angiogenesis [8,12,38,39]. Immediately after surgery the isolation chamber is filled with a non-viable fibrin matrix and therefore hypoxia is supposedly nonexistent in the chamber. However, cells, like pericytes, macrophages, and neutrophiles, migrate into the periphery [22] three days after loop implantation creating a hypoxic gradient [40]. Although hypoxia has a distinguished role in angiogenesis, it is most likely not the main factor influencing the vessel sprouting originating from the grafted vein in the AV loop model [41] and was also not observed in the AA model. Nevertheless, micro-CT analyses revealed directional sprouting from the surrounding tissue through the openings into the isolation chamber even in AA groups (Fig. 6C, D). It is tempting to speculate that this remaining angiogenesis that did not originate from the graft is triggered by a hypoxic gradient, which prevails in the isolation chamber after some days of implantation. Possibly blood flow (shear stress) increase is required for angiogenesis in response to hypoxic signaling which is in line with the observations from the developing zebrafish embryo [12].

Blood flow increased significantly immediately after interposition of the venous graft between femoral artery and vein. After 15 days blood flow through the loop was decreased again in the AV construct and attained levels comparable to untreated vessels. This alteration over time was observed previously and was explained by a pronounced increase of resistance along the grafted vessel generated by the newly formed 3-dimensional vessel network [19]. This view is supported by the fact that these new vessels were stained with injected ink indicative of their perfusion.

Recent studies focused on the role of the gap junction protein Cx43 in the cardiovascular system which exemplified its importance in angiogenesis [19,42]. Expression patterns in the rat aorta suggested that high flow or disturbed flow profile fosters the expression of Cx43 [43]. Indeed, enhanced flow load in the AV loop increased the level of Cx43 expression specifically in the venular endothelium at the protein as well as at the transcriptional level [19]. Physiologically, Cx43 protein is expressed only marginally in endothelial cells of the femoral vein. The current experiments demonstrate that mRNA levels of Cx43 were even decreased in AA grafted veins 5 days after loop construction, which is in marked contrast to the situation in veins used in AV shunts (Fig. 7). In vitro studies further suggested that cell stretch elicited by mechanical strain may also alter Cx43 expression, preferentially in smooth muscle cells [29]. However, mechanical strain due to enhanced transmural pressure and also flow pulsatility are comparable in AV and AA models. Therefore, we conclude that stretch and tension is not accountable for enhanced Cx43 expression in endothelial cells of the grafted vein in AV conditions [19] since Cx43 expression is strongly reduced in AA grafted veins (Fig. 7). Interestingly, the decreased mRNA expression of Cx43 and the lack of its upregulation was correlated with the absence of angigenesis in the AA model. This observation is compatible with the idea that the induction of Cx43 is a required step in shear stress induced angiogenesis.

## Conclusion

In summary, we identified in the current study the decisive role of flow increases for new vessel formation in a well established *in vivo* model (AV shunt) for hemodynamically driven angiogenesis [19,41]. By comparing AV loops with arterioarterial loops (AA) we demonstrate that introduction of pulsatility and exposure to arterial pressure levels is not sufficient to initiate angiogenetic sprouting from a grafted vein. While flow increase is decisive it remains to be determined if flow increase is sufficient in its own right or in response to hypoxic signaling. This interesting model will allow further evaluation of the endothelial response to enhanced wall shear stress *in vivo*. Angiogenesis derived from an AV shunt may be a useful strategy to support survival of tissue grafts.

## Supporting Information

S1 Video 3-dimensional micro-CT video of an AA loop. Luminal sprouting was absent along the grafted vein. However, directional sprouting from the surrounding tissue through the openings into the isolation chamber was evident.
(AVI)

S2 Video Micro-CT video of an AA loop demonstrating the absence of angiogenesis along the venous graft. (AVI)

S3 Video 3-dimensional micro-CT video of an AV loop. Homogeneous and strong luminal sprouting is present along the grafted vein.
(AVI)
